# Developmental biology and substrate preference of the quarantine pest, mango pulp weevil *Sternochetus frigidus* (Coleoptera: Curculionidae) under laboratory conditions

**DOI:** 10.1371/journal.pone.0345209

**Published:** 2026-10-05

**Authors:** Sayesta Mehejaveen Begum, Badal Bhattacharyya, Shimantini Borkataki, Kanchan Saikia, Bikash Hazarika, K. Sindhura Bhairavi, Shareen Tikhak, Ritu Ranjan Taye, Inee Gogoi, Supriya Sharma, Saurabh Sarma

**Affiliations:** 1 Department of Entomology, College of Agriculture, Assam Agricultural University, Jorhat, Assam, India; 2 Regional Research Station, ICAR-Indian Agricultural Research Institute-Assam, Kamrup, Assam, India; 3 Department of Horticulture, College of Agriculture, Assam Agricultural University, Jorhat, Assam, India; 4 AAU-Zonal Research Station, Sribhumi, Assam, India; 5 AICRP on Fruits, Assam Agricultural University, Jorhat, Assam, India; 6 Department of Plant Pathology, College of Agriculture, Assam Agricultural University, Jorhat, Assam, India; 7 Indian Council of Agricultural Research-Krishi Vigyan Kendra Hailakandi, ICAR RC for NEH Region, Hailakandi, Assam, India; Tea Research Association, INDIA

## Abstract

The mango pulp weevil, *Sternochetus frigidus* (Coleoptera: Curculionidae), is a major monophagous and quarantine pest of mango (*Mangifera indica*) in tropical Asia, causing severe yield and quality losses due to its concealed feeding and development within fruit tissues. A laboratory study was conducted to investigate the developmental biology, morphometrics and substrate preference of *S. frigidus* using seven mango substrates: young flush, old flush, green fruit (whole and sliced), ripened fruit (whole and sliced) and stem. Experimental rearing recorded all post-ovipositional stages except the egg phase due to lack of suitable specimens and the weevil completed five larval instars with a larva to adult period of 36–47 days across substrates without significant variation. Larvae were apodous and yellowish, reaching up to 14–15 mm in length, while pupae were exarate, measuring approximately 8.8 mm in length. Adult females exhibited larger abdominal dimensions and longer antennae than males, indicating clear sexual dimorphism. Feeding and aggregation studies revealed a strong preference for sliced ripened mango fruit, followed by sliced green fruit, suggesting that exposed fruit tissues and associated volatiles strongly influence adult attraction. Infested fruits consistently showed symptoms of anthracnose, with *Colletotrichum gloeosporioides* identified from affected tissues, indicating that weevil feeding facilitates secondary fungal infection. The findings provide essential biological and behavioural insights into *S. frigidus* that are directly relevant to mass rearing, detection and the development of sustainable management strategies for mango production and quarantine protection.

## Introduction

Mango (*Mangifera indica*) is one of the most important tropical fruit crops, widely cultivated and traded across Asia, Africa and Latin America. In mango-growing regions, insect pests pose a major constraint to productivity, fruit quality and international market access. Among these, the mango pulp weevil, *Sternochetus frigidus* Fabricius (Coleoptera: Curculionidae), is regarded as one of the most destructive and difficult-to-manage pests due to its concealed larval development within mango fruit tissues. The cryptic nature of infestation not only results in direct yield and quality losses but also creates serious challenges for detection, quarantine inspection and effective control [1].

*Sternochetus frigidus* is a monophagous pest restricted to mango and is widely distributed in South and South East Asia. Infestation begins when adult females deposit single eggs on the surface of young fruits and seal them with a waxy exudate. Upon hatching, larvae penetrate the fruit pulp and complete development through five larval instars within the fruit [1,2]. Infestations can reduce yields drastically with documented orchard losses ranging between 30 and 80% [2]. Because of the hidden nature of larval feeding, external symptoms are rare before emergence, complicating detection and management often resulting in rejected consignments and trade restrictions. The mango pulp weevil *Sternochetus frigidus* is recognised as a major concealed pest of mango and is of particular concern due to its quarantine significance and association with post-harvest losses [3]. Previous studies have highlighted that larval development occurs almost entirely within the fruit, resulting in infestations that frequently remain undetected until fruit ripening or processing. Adult behaviour, particularly attraction to host fruit cues, plays a critical role in infestation dynamics, yet experimental information on substrate-based responses and developmental plasticity under controlled conditions remains limited [3]. Effective management of concealed fruit-infesting weevils relies heavily on a sound understanding of their developmental biology, behaviour and host-use patterns. Information on developmental duration, number of larval instars, sexual dimorphism and feeding or aggregation preferences is essential for identifying vulnerable life stages, optimising monitoring tools and improving the timing of management interventions. In addition, laboratory-based biological studies provide critical baseline data for mass rearing, host susceptibility assessments and the development of detection or attractant-based strategies.

A comprehensive grasp of the biology and ecology of *S. frigidus* is crucial, given its status as a destructive mango pest. However, knowledge of its life cycle remains fragmentary with scant detail on fecundity, survival across instars and responses to ecological factors. Laboratory-based investigations are therefore essential to determine its developmental limits, longevity and morphometric characterisation under controlled conditions. Such bioecological data would provide a baseline for population models and management strategies. Accordingly, this research examines the bioecology of *S. frigidus* in controlled laboratory settings with emphasis on its development, survival and adult longevity. The present study was therefore conducted to investigate the developmental biology, morphometric characteristics and substrate preference of *S. frigidus* under laboratory conditions. In particular, seven mango substrates were chosen to represent the diversity of plant tissues accessible to mango pulp weevil under orchard conditions, including flush at different stages, fruits in both intact and sliced states and stem tissue. Flush tissues were selected based on observed adult feeding during early growth stages whereas fruits are recognised as primary sites for nutrition and oviposition. Stem tissue was included to examine whether development can occur within more rigid host structures. Collectively, these substrates facilitate evaluation of how developmental success and aggregation vary between vegetative and reproductive host parts. By examining larval and pupal development, adult sexual dimorphism and feeding responses to different mango substrates, this study aims to generate biologically relevant information that can support improved pest detection, laboratory rearing and the development of sustainable management strategies for mango production and quarantine protection. The findings will provide fundamental data necessary for understanding its population dynamics and supporting future management strategies.

## Materials and methods

### Test fruit

A widely cultivated local mango cultivar from Assam was selected for this study based on its availability, known susceptibility to *Sternochetus frigidus* and importance in local cultivation systems during 2023−25. Seven mango plant substrates were evaluated: young flush, old flush, whole green fruit, sliced green fruit, whole ripened fruit, sliced ripened fruit and stem.

### Insect collection and laboratory culture

Infested mango fruits were collected from mango trees in and around Assam Agricultural University, Jorhat campus including Experimental Farm, Department of Horticulture and Department of Animal Husbandry and Dairying and transferred to the Chemical Ecology Laboratory, Department of Entomology for insect recovery. Adult *Sternochetus frigidus* emerging from infested fruits were used to establish a laboratory culture. The culture was maintained in rearing trays at 27 ± 2 °C, 70 ± 5% relative humidity and a 12:12 h (L:D) photoperiod. These conditions were selected to approximate mean temperature and humidity during the main mango fruiting period in Jorhat (Assam) and fall within the range previously used for successful laboratory rearing of *Sternochetus* species on mango fruits [4,5]. Fresh mango substrates were provided as food and oviposition sources and replaced at regular intervals to maintain hygiene and prevent microbial contamination.

### Developmental biology

Eggs laid on mango fruits by *S. frigidus* are characteristically sealed with a waxy layer and therefore collection was not possible in both field-collected and laboratory-reared populations because they often hatched before they could be retrieved. Despite repeated attempts, eggs could rarely be obtained intact in sufficient numbers for reliable developmental or morphometric measurements and hence were therefore excluded from the developmental biology as well as morphometric analysis. Developmental observations therefore commenced from larva and the durations reported in this study refer to post‑ovipositional stages from larva to adult emergence. Larval development was monitored daily and the number of instars was determined based on moulting frequency and head capsule measurements. Duration of each larval instar, pupal period and total development time from larvae to adult emergence were recorded under laboratory conditions.

### Morphometric analysis

Larval specimens used for morphometric analysis were obtained from mango fruits that were either naturally infested in the field or exposed in the laboratory to adults morphologically identified as *S. frigidus*. For each larval cohort, a subsample was reared through to adult emergence and only cohorts that yielded adults conforming to published descriptions and diagnostic characters for *Sternochetus frigidus* on mango were retained for analysis. The adult morphology of *S. frigidus* was thoroughly studied by careful dissection and detailed observation under Zeiss Axiocam ERc 5s stereomicroscope and detailed morphometric evaluations of the requisite structures were performed using Carl Zeiss Stemi 508 Trinocular stereomicroscope in 0.65x magnification. Similarly, the morphometric attributes of the larval and pupal stages were meticulously studied under Carl Zeiss Stemi 508 Trinocular stereomicroscope in 0.65x magnification, with data recorded for key structural parameters including body length and breadth. Sex was determined based on external morphological characteristics and measurements of males and females were compared to assess sexual dimorphism. Detailed morphometric images and measurements are provided in result section.

### Substrate preference assay

Adult substrate preference was assessed using seven mango substrates: young flush, old flush, whole green fruit, sliced green fruit, whole ripened fruit, sliced ripened fruit and mango stem. Individual substrates were arranged equidistantly in plastic containers and adult weevils were released at the centre. The number of adults attracted to each substrate was recorded at regular intervals up to 36 hours. Each treatment was replicated thrice under controlled laboratory conditions.

### Fungal association

Mango fruits exhibiting symptoms of decay following *S. frigidus* infestation were examined for associated fungal pathogens. Infected tissues were surface sterilised and cultured on potato dextrose agar. Fungal isolates were identified based on colony morphology and microscopic characteristics using standard mycological procedures of *Colletotrichum gloeosporioides* associated with mango anthracnose [6,7].

### Statistical analysis

Descriptive statistics were calculated for developmental duration, morphometric parameters and aggregation counts. Differences among treatments were analysed using one-way analysis of variance (ANOVA), followed by Tukey’s honestly significant difference (HSD) test at α = 0.05.

## Results

### Developmental biology of *Sternochetus frigidus* on different substrates

All post-ovipositional developmental stages of *Sternochetus frigidus* were successfully reared on each of the seven mango substrates under laboratory conditions. Developmental progression was consistent across substrates, indicating limited influence of substrate type on developmental chronology. Although immature and adult stages were observed, the egg stage could not be included due to the inability to obtain suitable specimens under laboratory rearing conditions. The total developmental duration from larval hatch to adult emergence ranged from 36 to 47 days, with no statistically significant differences among substrates (Table 1). This similarity in development time across young flush, fruit tissues and stem suggests that under controlled conditions, all evaluated mango parts provide nutritionally adequate resources for completion of immature development, despite their contrasting structure and maturity. Mean developmental periods for individual life stages across treatments are presented in Table 1. The concealed completion of development within the fruit observed in the present study is consistent with earlier reports that emphasise the cryptic nature of *S. frigidus* infestation and the associated challenges for early detection and management [3].

**Table 1:** Developmental duration of *Sternochetus frigidus* across substrates under laboratory conditions.

Treatment	Duration of developmental stages (in days)						
	1^st^ instar	2^nd^ instar	3^rd^ instar	4^th^ instar	5^th^ instar	Pupa	Adult longevity
Young mango flush	5.67 ± 0.33	6.67 ± 0.58	8.33 ± 0.58	6.67 ± 0.58	6.33 ± 0.58	10.33 ± 0.33	6.67 ± 2.03
Old, hardened mango flush	6.33 ± 0.88	7.33 ± 0.67	8.33 ± 1.52	6.67 ± 1.15	6.33 ± 1.53	10.00 ± 0.58	9.33 ± 0.67
Green mango fruit (whole)	6.33 ± 0.67	7.67 ± 0.58	8.67 ± 0.58	6.00 ± 1.00	6.67 ± 0.58	9.67 ± 0.33	6.67 ± 2.62
Green mango fruit (sliced)	5.33 ± 0.33	6.67 ± 0.33	8.67 ± 1.53	6.33 ± 0.58	5.67 ± 0.58	9.67 ± 0.67	4.00 ± 1.00
Ripened mango fruit (whole)	5.33 ± 1.67	7.00 ± 0.94	8.33 ± 0.52	6.33 ± 1.53	7.00 ± 1.00	10.00 ± 0.58	4.00 ± 2.31
Ripened mango fruit (sliced)	6.00 ± 0.58	7.33 ± 0.88	8.00 ± 1.00	6.33 ± 0.58	6.67 ± 1.15	9.67 ± 0.33	9.33 ± 4.81
Stem	6.00 ± 0.58	7.33 ± 0.33	8.00 ± 1.00	5.67 ± 0.58	6.67 ± 0.53	10.67 ± 0.33	6.33 ± 1.45
F	1.286	0.570	0.331	0.500	0.792	0.792	1.120
*p*-value (<0.05)	0.315	0.751	0.911	0.800	0.590	0.590	0.396

Values are expressed as mean ± SD (n = 3) with 30 samples each.

### Morphological parameters of immature stages

Larvae of *Sternochetus frigidus* were legless, yellowish and wrinkled which increased steadily in size through five instars attaining about 14–15 mm in length and a width of 3–4 mm (Table 2). The head capsule was well sclerotised with prominent mouthparts and successive larval instars were distinguished by progressive increases in body length and head capsule width.

**Table 2:** Morphometric parameters of developmental stages of *Sternochetus frigidus* under laboratory conditions.

Sl. No.	Developmental Stages	Mean (mm) ± SD	
		Length	Breadth
I.	Larva		
a.	First instar	1.70 ± 0.06	0.36 ± 0.02
b.	Second instar	4.50 ± 0.43	0.52 ± 0.05
c.	Third instar	7.03 ± 0.07	1.33 ± 0.06
d.	Fourth instar	10.39 ± 0.41	1.84 ± 0.06
e.	Fifth instar	14.16 ± 0.85	3.17 ± 0.40
II.	Pupa	8.84 ± 0.04	4.92 ± 0.07

Values are expressed as mean ± SD (n = 15).

Pupae were exarate and developed within loosely formed chambers constructed from frass inside the fruit pulp with mean length around 8.84 ± 0.04 mm and width 4.92 ± 0.07 mm (Table 2). Sexual dimorphism was not clearly evident at the pupal stage, although females exhibited slightly greater abdominal width.

### Morphological parameters of adults

Adult *Sternochetus frigidus* exhibited clear sexual dimorphism in several morphometric traits (Table 3). Males were marginally longer, whereas females possessed more enlarged abdomens with significantly greater length and width, reflecting their reproductive differentiation. Female adults also possessed longer antennae than males while snout length and elytral measurements showed no marked variation between sexes. All adults exhibited uniform tetramerous tarsal segmentation across the three pairs of legs.

**Table 3:** Morphometric characters of adult *Sternochetus frigidus* under laboratory conditions.

Sl. No.	Characteristics	Adult Mean (mm)±SD	
		Male	Female
1.	Body		
a.	Head Length (HL)	1.65 ± 0.08	1.62 ± 0.06
b.	Head Width (HW)	1.74 ± 0.05	1.69 ± 0.02
c.	Thorax Length (TL)	2.19 ± 0.04	1.87 ± 0.02
d.	Thorax Width (TW)	1.92 ± 0.03	1.91 ± 0.03
e.	Abdomen Length (AL)	1.70 ± 0.06	1.94 ± 0.02
f.	Abdomen Width (AW)	1.54 ± 0.02	1.87 ± 0.01
g.	Body Length (BL)	3.24 ± 0.06	3.01 ± 0.05
h.	Body Width (BW)	1.85 ± 0.06	1.70 ± 0.03
2.	Antennal Length (AnL)	3.85 ± 0.19	4.58 ± 0.33
3.	Snout Length (SnL)	3.52 ± 0.08	2.88 ± 0.17
4.	Elytra Length (EL)	3.91 ± 0.08	3.95 ± 0.14
5.	Elytra Width (EW)	2.63 ± 0.04	2.76 ± 0.09
6.	Pygidial Apex (PA)	Not visible	Not visible
7.	Legs		
a.	Foreleg Length (FLL)	6.59 ± 0.23	6.72 ± 0.42
b.	Foreleg Width (FLW)	1.52 ± 0.11	1.20 ± 0.11
c.	Mid Leg Length (MLL)	6.32 ± 0.08	5.32 ± 0.15
d.	Mid Leg Width	1.39 ± 0.09	1.09 ± 0.13
e.	Hind Leg Length (HL)	6.36 ± 0.11	5.96 ± 0.14
f.	Hind Leg Width (HLW)	1.57 ± 0.12	1.59 ± 0.06
g.	Tarsal Segments	4	4

Values are expressed as mean ± SD (n = 15).

Sexual dimorphism was further evident in thoracic morphology, with females showing a more convex ventral thorax compared with the relatively flattened thorax of males. Male genital structures, including overlapping apical sclerites of the aedeagus were consistent with species-level diagnostic characteristics. Detailed morphometric measurements of male and female adults are presented in Table 3, while representative images are provided as Supplementary material.

### Aggregation of weevils on different substrates

Adult *Sternochetus frigidus* exhibited differential aggregation across the mango substrates evaluated under laboratory conditions. Aggregation responses were recorded at 6 hour intervals up to 36 hours, and the mean number of adults associated with each substrate is presented in Table 4. The results indicate clear substrate-specific aggregation patterns among the tested mango plant parts. Previous study suggested that adult *S. frigidus* respond strongly to host-derived cues, particularly volatiles emitted from mango fruit [3]. The pronounced aggregation observed on sliced green and ripened mango fruits in the present study supports this view and provides experimental evidence that exposed fruit tissues enhance adult attraction. Such substrate-specific responses highlight the potential for exploiting fruit-based cues in the development of improved monitoring tools and attract-and-kill strategies.

**Table 4:** Aggregation of adult *Sternochetus frigidus* on distinct mango substrates over different time intervals.

Treatment	6h	12h	18h	24h	30h	36h
Young mango flush	7.33 ± 1.52^c^	8.00 ± 3.00^c^	7.33 ± 3.05^b^	6.33 ± 3.05^c^	7.66 ± 3.78^c^	8.66 ± 2.08^d^
Old, hardened mango flush	6.66 ± 1.52^c^	7.00 ± 1.00^c^	4.00 ± 1.00^c^	4.00 ± 1.00^d^	3.66 ± 4.00^d^	3.33 ± 1.52^e^
Green mango fruit (whole)	4.33 ± 1.52^d^	6.00 ± 1.00^c^	8.33 ± 1.52^ab^	8.33 ± 2.08^b^	6.00 ± 1.00^d^	5.33 ± 1.52^c^
Green mango fruit (sliced)	13.66 ± 3.05^b^	9.66 ± 1.52^b^	10.33 ± 4.16^a^	11.66 ± 2.08^a^	14.33 ± 2.51^d^	20.66 ± 1.52^a^
Ripened mango fruit (whole)	11.33 ± 1.52^b^	14.66 ± 3.21^a^	10.00 ± 1.00^a^	9.00 ± 3.00^b^	10.00 ± 3.00^b^	15.66 ± 1.52^b^
Ripened mango fruit (sliced)	18.00 ± 2.64^a^	20.33 ± 6.65^a^	14.66 ± 4.16^a^	18.33 ± 7.02^a^	16.00 ± 4.58^a^	17.00 ± 5.56^b^
Stem	3.33 ± 0.57^e^	3.33 ± 1.52^d^	4.66 ± 1.15^c^	5.66 ± 0.57^d^	3.66 ± 1.15^d^	3.66 ± 1.52^e^
C.D	3.37	5.89	4.66	5.83	4.83	4.53
*p*-value (<0.05)	0.001	0.001	0.003	0.002	0.001	0.001

Data represented is the mean of 3 replications with 30 samples each. Means followed by the same letter within a column are not significantly different according to Tukey’s HSD test at P = 0.05.

One-way analysis of variance conducted at each observation interval indicated significant differences in adult aggregation among the seven mango substrates (P ≤ 0.001). Sliced ripened mango fruit consistently attracted the highest number of adults from the initial 6h and maintained this preference throughout the 36h observation period. In contrast, aggregation on sliced green mango fruit increased progressively over time, indicating sustained attraction under laboratory conditions.

### Onset of secondary infection by pathogen due to weevil activity

In the aftermath of aggregated feeding by *S. frigidus*, several mango fruits developed evident signs of secondary microbial infections which were visually identifiable by darkened lesions resembling anthracnose. Notably during field collection for insect screening, mangoes harbouring *S. frigidus* larvae as well as adults consistently manifested anthracnose symptoms whereas adjacent fruits from the same tree, devoid of infestation remained externally pristine and lesion-free. Further, sections of necrotic tissue excised from affected mangoes were subjected for fungal identification using standard mycological mounting protocols. The spores observed are primarily oblong to cylindrical with smoothly rounded extremities, hyaline, aseptate and morphologically consistent across the field of view. The conidia were dispersed singly without septation or pronounced curvature thereby distinguishing them from other *Colletotrichum* species exhibiting falcate or highly curved spores. These morphological characteristics, together with the typical anthracnose symptoms observed on mango fruits, confirmed *Colletotrichum gloeosporioides* (Phylum: Ascomycota; Class: Sordariomycetes; Family: Glomerellaceae) as the associated pathogen. This discernible association plausibly indicated that the covert endophytic activity and internal feeding by *S. frigidus* may predispose mango fruits to opportunistic fungal colonisation.

## Discussion

### Laboratory rearing of *Sternochetus frigidus*

All post‐ovipositional stages were reared on seven mango substrates. The overall developmental time from larva to adult ranged about 36–47 days. Statistical analysis confirmed no significant differences among substrates. The consistency in the development period is to be expected, considering the strict monophagy of the pest on mango and the substrates used which likely possessed similar nutritional properties. The observed development period in this study is broadly consistent with earlier findings. Researchers found a complete life cycle of around 32 days in Carabao mango, which is slightly shorter than our average [4]. Other researchers recorded development within fruit to take around 36.7 days which aligned with the timeframe of the recorded data [8]. Researchers also documented a longer cycle of 50 days in *S. mangiferae*, a different species of the same genus, noting that such differences may arise from climatic or varietal influences [9]. A notable comparison can be drawn with *S. gravis* for which researchers observed a life cycle lasting for 58–60 days under laboratory conditions [5]. In contrast, the present study showed that *S. frigidus* required only 36–47 days in reaching maturity. The difference may be attributed to species-specific biology or varying rearing conditions. Despite this variation, both species exhibited a common trait in completing their development within the fruit itself which strengthens the view that *Sternochetus* weevils are finely tuned to the seasonal cycle of their host. The occurrence of pupation inside the pulp chamber in the present study corresponded with the findings of researchers [10] for *S. frigidus* and [5] for *S. gravis* where pupation was also recorded inside the fruit. Overall, the results reveal a consistent developmental rhythm in *S. frigidus* that corresponded with prior research. Slight differences in duration are likely due to differences in mango cultivar, climate conditions or rearing environment. Moreover, no substrate emerged as notably better or worse despite their diversity. For a strictly monophagous species such as *S. frigidus* [1–4], this pattern appears ecologically plausible as larvae are evolutionarily adapted to complete development within mango tissues and may therefore display resilience to intra-host variability [11,12]. Despite differences in moisture, fibre content and secondary metabolites among young flush, fruit and stem, their shared origin ensures a comparable nutritional framework especially under the non‑limiting, low‑stress conditions of the laboratory. Behavioural modulation in feeding may mitigate nutritional differences, producing comparable developmental durations. Field experiments that expose larvae to naturally fluctuating humidity, temperature and fruit load would be valuable to test whether this apparent developmental homeostasis persists under more heterogeneous nutritional conditions.

### Morphometric variation across developmental stages and sexual dimorphism in *Sternochetus frigidus*

The morphometric measurements of both immature and adult stages largely corresponded with existing publications, with a few notable points of divergence. The fifth instar larvae attained an average length of 14.16 ± 0.85 mm and a breadth of 3.17 ± 0.40 mm consistent with the observations reported by researchers [8] who observed fifth instars of *S. frigidus* exceeding 1 cm and other researchers [13] who reported mean values of 14.78 mm in length and 2.57 mm in width. A near match in size was observed for *S. gravis*, whose fifth instars averaged 14.04 ± 0.17 mm long and 3.29 ± 0.12 mm breadth [5]. Larval features including the head capsule and the distinctive curved, legless body corresponds closely with descriptions provided by researchers [14,15] who described similar pulp-feeding grubs. Pupal measurements recorded in this study measured on average 8.84 ± 0.04 mm in length and 4.92 ± 0.07 mm in breadth. These measurements are remarkably similar to those recorded for *S. gravis* by Devi *et al.* (2011) who reported similar dimensions of 8.81 ± 0.30 mm length and 4.94 ± 0.17 breadth suggesting a strong similarity in pupal form across species reared in laboratory conditions. In contrast, researchers found notably smaller pupae, averaging around 8.06 ± 0.28 mm by 4.27 ± 0.046 mm [13], while other researchers recorded estimates of 8 mm in length and 4 mm in breadth for *S. frigidus* [8]. The slightly larger size observed in the pupae could be resulted from the choice of mango, the feeding conditions or variation across regional strains. No clear sexual differences were evident during the study.

Adult weevils in the present study measured approximately 3.24 ± 0.06 mm in length and 1.85 ± 0.06 mm in breadth for males while females averaged 3.01 ± 0.05 mm in length and 1.70 ± 0.03 mm in breadth. These measurements are modest compared to the 5–8 mm range reported by researchers for *S. frigidus* [15]. They are however within the lower margin of the 3.8 to 5.9 mm span as per earlier report [16]. Such variation may arise from measurement techniques particularly whether the snout was included or from regional differences in population size.

A clear distinction between the sexes was evident in the present data. Female weevils displayed noticeably longer abdomens averaging about 1.94 ± 0.02 mm compared to 1.70 ± 0.06 mm. This elongation was accompanied by greater abdominal width in females, averaging 1.87 ± 0.01 mm compared to 1.54 ± 0.02 mm in males suggesting a morphology favouring increased egg-carrying potential, an interpretation that resonated with a convex ventral thorax in females outlined in the work of Balock and Kozuma (1964) [17] and subsequently reaffirmed by Syahputra *et al.* (2019) [18] whereas the male morphology characteristically displayed a flatter ventral surface. The male specimens examined revealed traits long used in identifying *S. frigidus,* notably the absence of a median lobe on the eighth tergite as described by De Jesus (2002) [18] along with overlapping apical sclerites in the aedeagus, a character consistent with observations by Syahputra *et al.* (2019) [8] and Euatrakool (2015) [19] as a reliable means of distinguishing *S. frigidus* from other species. Tarsal segmentation remained consistent across sexes, with each leg bearing four segments a trait typical of Curculionidae in line with the morphological norms on standard curculionid morphology reported by Legalov (2020) [20] and Marvaldi and Lanteri (2005) [21]. Overall, the present morphometric findings largely reinforced earlier findings while adding nuance upon earlier work. Larval and pupal dimensions were consistent with those noted by Syahputra *et al.* (2019) [8] and Bhattacharyya *et al.* (2024) [13] while adult sizes showed more compact proportions similar to the lower thresholds described [16]. Sexual dimorphism observed in the shape of the ventral thorax aligned with established descriptions [17,18]. The modest variation we recorded might be attributed to natural diversity among weevil populations or variation brought on by the rearing conditions.

### Antennal structure

The present research examined the antennal morphology and noted that female weevils exhibited slightly longer antennae, with mean filament length around 4.58 ± 0.33 mm compared to 3.85 ± 0.19 mm in males. Both males and females exhibited a nine segmented geniculate antenna wherein the filament was structured with six funicular segments followed consecutively by a compact three-segmented club widely recognised as typical characteristic of Curculionidae. This structure aligned with those described in other curculionid subfamilies as noted by Lyal (1993) [22]; Anderson (2002) [23] and Marvaldi and Lanteri (2005) [21] highlighting a stable morphological trait within the family. As far as knowledge goes, the present research would be the first quantified report of antennal length difference in *S. frigidus*. Female antennae may be longer to accommodate a denser array of olfactory sensors and this feature is frequently observed in sexually dimorphic insect species. This pattern was plausible since females in many insect groups are known to possess more specialised olfactory systems for host detection and oviposition, as outlined [24]. Bichão *et al.* (2005) [25] reported sex-specific antennal neurons in a related weevil species, *Anthonomus rubi*, commonly known as strawberry blossom weevil. While this study did not include electrophysiological tests, the antennal structure in females hinted at greater sensitivity to pheromones or host volatiles. This was particularly relevant since De Jesus (2008) [26] observed virgin females responding intensely to male-emitted volatiles. Altogether, antennal structure in our specimens reflected classic weevil features with filament length emerging as the main difference between sexes. This variation may support the role of female in finding oviposition sites though upcoming behavioural tests would offer clearer insight into any sex-linked olfactory function.

### Feeding preference on different mango substrates

The present study also examined the feeding preference of *S. frigidus* on seven mango substrates through controlled laboratory trials. Perusal of data presented in the findings indicated that *S. frigidus* adults strongly preferred mango fruit tissues over other substrates as evidenced by their high aggregation on sliced ripened mango fruit and on sliced green mango fruit. This behaviour was consistent with the frugivorous pattern of mango pulp weevil as noted by researchers [27] who noticed that adults feed on mango flowers and shift their activity towards green fruits during peak fruiting periods. Dhang (2015) [1] observed that adults feed by piercing the skins of developing mango fruits and their activity remained confined to mango fruits alone regardless of variety. These findings aligned closely with the present study wherein *S. frigidus* was predominantly attracted to the edible fruit portions while showing minimal activity on vegetative parts. By experimentally demonstrating substrate-specific adult aggregation and the association between infestation and secondary fungal infection, the present study extends previous knowledge on *S. frigidus* biology and provides applied insights relevant to monitoring, quarantine inspection and integrated pest management programmes [3].

Substrate preference in *S. frigidus* seemed to be mediated by fruit volatiles. As reported by De Jesus (2008) [26], adult *S. frigidus* responded strongly to volatiles emitted from green mango fruits indicating that fruit volatiles serve as host-location cues [3]. In the present experiment, the sliced ripened mango fruit might have likely emitted higher concentrations of such volatiles due to exposed tissues which may explain the highest aggregation of weevils. Similarly, the sliced green mango fruit drew a steady increase in weevil aggregation across time intervals possibly due to a mixture of structural integrity and chemical profile. The limited feeding and aggregation levels observed on vegetative treatments further supported the hypothesis that *S. frigidus* was adapted to fruit consumption. These substrates might have likely lacked the chemical or structural cues required to initiate and sustain feeding activity. Similar conclusions have been drawn in earlier research where Dhang, (2015) [1] reported this pattern of feeding preference of *S. frigidus* over pulp while avoiding non-fruit parts when fruits were available. In conclusion, the present findings revealed that *S. frigidus* adults were most strongly drawn towards ripened and green mango fruits with significantly higher aggregation in these treatments. These findings were consistent with published literature on host selection by *S. frigidus* and validated the involvement of mango fruit volatiles and physical traits in mediating feeding preferences.

### Secondary infection and pathogen interactions

Experimental findings of the present study revealed a clear and consistent association between *S. frigidus* infestation and the development of anthracnose lesions in mango fruits. Mangoes that harboured *S. frigidus* adults or larvae invariably exhibited external lesions that were dark, sunken and irregularly scattered across the fruit surface, morphologically characteristic of anthracnose caused by *Colletotrichum gloeosporioides*. Microscopic examination of necrotic tissue sections confirmed the identity of the pathogen marked by the presence of hyaline and aseptate conidia that were oblong to cylindrical in shape ending with smooth rounded tips thus validating it as *C. gloeosporioides* as described in earlier studies [16,17]. These fungal structures were exclusively isolated from fruits infested by weevils, thereby strengthening the association between the feeding behaviour of *S. frigidus* and pathogen invasion. The most reasonable cause lies in the structural breaches inflicted by weevil activity. As both adults and larvae penetrated and fed on the mango pulp, they damaged the outer and inner fruit layers rendering the fruit susceptible for fungal entry. These breaches acted as direct entry points for fungal spores to colonise the tissue. Jenny *et al.* (2019) [28] emphasized that anthracnose lesions proliferated faster on wounded mango tissues providing a moist and nutrient rich environment for *Colletotrichum* conidial germination. In this context, the results of the present study affirmed that feeding by *S. frigidus* effectively predisposes mango fruit to opportunistic fungal invasion particularly by *C. gloeosporioides*. Similar associations between the activity of frugivorous insect and incidence of anthracnose have been reported in other tropical fruits, the explicit visual and microscopic corroboration in this study offered more definitive evidence for mango systems. However, *S. frigidus* does not act as a biological vector of the pathogen but facilitated infection as a mechanical disruptor that enabled fungal entry through its direct feeding thereby contributing to quality loss.

### Management implications

The present findings have direct implications for the management of *Sternochetus frigidus* in mango production systems. The consistent completion of larval and pupal development within the fruit pulp confirms that control measures targeting immature stages are unlikely to be effective once oviposition has occurred, reinforcing the need to focus management efforts on the adult stage. The strong and consistent aggregation of adults on sliced green and ripened mango fruits suggests that exposed fruit tissues and associated volatiles play a key role in host location and aggregation behaviour [3]. These responses could be exploited for the development of fruit-based attractants or baited monitoring systems to improve early detection of adult activity. Additionally, the observed association between weevil infestation and anthracnose symptoms indicates that *S. frigidus* infestation may exacerbate post-harvest losses by predisposing fruits to secondary fungal infection. Timely management of adult populations before fruit penetration may therefore contribute not only to reducing direct insect damage but also to limiting secondary disease development, with potential benefits for both yield protection and quarantine compliance.

### Limitations and future directions

Responses related to aggregation and feeding responses documented here were obtained under controlled laboratory conditions using confined choice arenas and a single local cultivar which do not replicate the variability inherent in orchard ecosystems [29,30]. Under field conditions, adults encounter fruits of varying age, competing odour sources and natural enemies. The strong preference for sliced ripened and sliced green fruits may be accentuated by the artificially high exposure of volatiles from cut surfaces and by the absence of alternative non‑fruit resources [1]. Hence, the observed aggregation should be regarded as a maximal response rather than a direct reflection of field dynamics. Validation through field experiments using intact fruits and naturally injured tissues would be valuable to validate whether similar substrate preferences and aggregation patterns occur under commercial orchard conditions.

## Conclusion

The present study provides comprehensive laboratory-based information on the developmental biology, morphology and substrate preference of the mango pulp weevil *Sternochetus frigidus*, a major concealed pest of mango. All post-ovipositional stages were successfully reared on different mango substrates and developmental duration remained consistent across substrates, indicating limited influence of host tissue variation on immature development. Completion of larval and pupal development entirely within the fruit pulp highlights the cryptic nature of infestation and the difficulty of detection once oviposition has occurred. Clear sexual dimorphism was observed in adult morphometric traits while larval development progressed through five instars with predictable morphological changes. Adult aggregation assays demonstrated strong and consistent preference for sliced green and ripened mango fruits, suggesting that exposed fruit tissues release cues that enhance host location and aggregation. The frequent association of *S. frigidus* infestation with anthracnose symptoms further indicates that weevil feeding predisposes mango fruits to secondary fungal infection, thereby compounding post-harvest losses. Collectively, these findings provide biologically relevant insights that can support improved laboratory rearing, refinement of monitoring and detection strategies and better timing of management interventions targeting adult weevils before oviposition. The information generated is particularly relevant for mango production systems and quarantine programmes aimed at reducing the economic and trade impacts of this pest.
